# Adherence to National Guidelines for Timeliness of Test Results Communication to Patients in the Veterans Affairs Health Care System

**DOI:** 10.1001/jamanetworkopen.2022.8568

**Published:** 2022-04-22

**Authors:** Ashley N. D. Meyer, Taylor M. T. Scott, Hardeep Singh

**Affiliations:** 1 Center for Innovations in Quality, Effectiveness and Safety, Michael E. DeBakey VA Medical Center and Department of Medicine, Baylor College of Medicine, Houston, Texas

## Abstract

This cross-sectional study assesses policy adherence to national guidelines for timeliness of test results communication to patients in the Department of Veteran Affairs health care system.

## Introduction

Failure to communicate test results to patients remains a persistent problem leading to diagnosis and management delays,^1,2,3^ with up to 62% of abnormal laboratory results and 36% of abnormal radiology results lacking timely follow up.^4^ The Department of Veterans Affairs (VA) developed a national policy in 2015 stating that practitioners authorized to order laboratory tests (referred to in the policy as *providers*), or their designees, must communicate abnormal test results to patients within 7 days if action is required and within 14 days if no action is required or results are normal.^5^ Acceptable communication modes include face-to-face, telehealth, telephone, secure messages, or letters. To assess policy adherence, the VA implemented a quality measurement system for feedback and improvement. We analyzed the first full year of these measures to determine timeliness of test results communication to patients.

## Methods

This cross-sectional study had VA institutional review board approval and was exempt from informed consent because no patient identifiable information was included in the data set. This study follows the Strengthening the Reporting of Observational Studies in Epidemiology (STROBE) reporting guideline. Data collection was implemented through the External Peer Review Program (EPRP), the VA’s performance measurement system that is used for quality improvement and benchmarking. Computerized algorithms randomly sample a set of abnormal and normal results related to 8 predetermined tests for each VA facility and each quarter (Table). Trained EPRP chart reviewers evaluated documentation of the communication of results to patients in medical records.

**Table.** Timeliness of Test Results Communication to Patients in the VA as Measured Through the EPRP^a^

Measure	Test results, No. (%)					Median (range), %^**b**^
	Quarter 1 (Oct-Dec 2018) (n = 4698)	Quarter 2 (Jan-Mar 2019) (n = 5161)	Quarter 3 (Apr-Jun 2019) (n = 5135)	Quarter 4 (Jul-Sep 2019) (n = 5162)	All quarters (Oct 2018-Sept 2019) (N = 20 156)	
EPRP^c^						
Abnormal test results^d^						
All	1506 (73.3)	1613 (68.0)	1435 (71.7)	1371 (70.5)	5925 (70.8)	71.4 (45.5-93.9)
AFP	221 (72.2)	186 (64.6)	193 (68.2)	210 (70.9)	810 (69.1)	
Chest CT	338 (76.0)	313 (70.8)	329 (75.3)	300 (72.3)	1280 (73.6)	
Chest radiograph	86 (87.8)	126 (83.4)	144 (85.2)	121 (85.2)	477 (85.2)	
DEXA	135 (63.7)	118 (58.4)	139 (67.1)	120 (58.0)	512 (61.8)	
FOBT	156 (76.1)	353 (67.8)	337 (69.8)	353 (71.3)	1199 (70.4)	
HCV	365 (69.5)	315 (62.4)	104 (60.1)	92 (62.2)	876 (64.8)	
Mammogram	129 (76.8)	122 (73.9)	120 (77.4)	118 (72.4)	489 (75.1)	
Papanicolaou test	76 (79.2)	80 (81.6)	69 (73.4)	57 (73.1)	282 (77.0)	
Normal test results^e^						
All	2178 (82.4)	2017 (72.3)	2595 (82.8)	2682 (83.3)	9472 (80.4)	81.3 (52.9-96.8)
AFP	201 (73.4)	162 (59.6)	195 (76.5)	193 (76.9)	751 (71.4)	
Chest CT	319 (82.2)	275 (69.8)	321 (80.5)	354 (83.3)	1269 (79.0)	
Chest radiograph	215 (87.4)	278 (82.7)	311 (92.0)	339 (93.4)	1143 (89.1)	
DEXA	424 (79.0)	361 (65.5)	432 (80.6)	458 (82.2)	1675 (76.8)	
FOBT	173 (66.3)	154 (55.8)	191 (74.0)	184 (71.3)	702 (66.7)	
HCV	144 (77.8)	115 (61.8)	424 (73.5)	399 (70.1)	1082 (71.3)	
Mammogram	78 (87.6)	70 (80.5)	79 (92.9)	88 (97.8)	315 (89.7)	
Papanicolaou test	624 (94.1)	602 (87.6)	642 (93.6)	667 (94.6)	2535 (92.5)	
All test results^f^						
All	3913 (83.3)	3967 (76.9)	4301 (83.8)	4298 (83.3)	16 479 (81.8)	82.3 (58.5-94.8)
AFP	468 (80.7)	397 (70.9)	431 (80.1)	442 (80.8)	1738 (78.1)	
Chest CT	701 (84.2)	655 (78.3)	705 (84.3)	702 (83.6)	2763 (82.6)	
Chest radiograph	310 (90.1)	416 (85.4)	461 (90.9)	467 (92.5)	1654 (89.7)	
DEXA	591 (78.9)	535 (71.0)	605 (81.4)	612 (80.1)	2343 (77.9)	
FOBT	343 (73.6)	570 (71.5)	565 (76.2)	575 (76.4)	2053 (74.5)	
HCV	558 (78.6)	479 (69.3)	591 (78.8)	540 (75.3)	2168 (75.6)	
Mammogram	219 (85.2)	198 (78.6)	205 (85.4)	216 (85.4)	838 (83.6)	
Papanicolaou test	723 (95.3)	717 (91.3)	738 (94.6)	744 (95.0)	2922 (94.0)	
SHEP^g^						
Patient response, No.	53 901	54 158	54 456	50 949	213 464	
Never	4251 (7.9)	4408 (8.1)	4538 (8.3)	4238 (8.3)	17 435 (8.2)	7.6 (2.6-23.5)
Sometimes	3307 (6.1)	3378 (6.2)	3565 (6.5)	3206 (6.3)	13 456 (6.3)	6.3 (1.8-10.9)
Usually	8502 (15.8)	8601 (15.9)	8658 (15.9)	7912 (15.5)	33 673 (15.8)	16.1 (10.6-22.5)
Always	37 841 (70.2)	37 771 (69.7)	37 695 (69.2)	35 593 (69.9)	148 900 (69.8)	69.7 (50.7-83.5)

Abbreviations: AFP, α-fetoprotein tests; CT, computed tomography; DEXA, dual-energy x-ray absorptiometry; EPRP, External Peer Review Program of the US Department of Veterans Affairs; FOBT, fecal occult blood screens; HCV, hepatitis C virus; SHEP, Survey of Healthcare Experiences of Patients in the US Department of Veterans Affairs; VA, US Department of Veterans Affairs.

^a^ This research includes data obtained from the Veteran Health Administration Office of Performance Measurement (17API2), which resides within the Office of Analytics and Performance Integration, under the Office of Quality and Patient Safety.

^b^ The EPRP median (range) included facility variation across all quarters.

^c^ For most facilities each quarter, computerized algorithms sample 6 random test results (4 abnormal and 2 normal) for each of the 8 test types listed in the table. However, for 9 of the largest facilities, computerized algorithms sample 9 random test results (6 abnormal and 3 normal) for each of the 8 test types listed.

^d^ Communicated in 7 days or less if action was required or 14 days or less if no action was required (n = 8372). Abnormal results were defined as AFP (>20 ng/mL); chest CT (verified abnormal per radiology code), chest radiograph (verified abnormal per radiology code); DEXA (T-score of −2.5 or lower); FOBT (positive screen); HCV (positive or reactive); mammogram (BI-RAD code 0, 3, 4, 5, or 6); and Papanicolaou tests (atypical squamous cells, low grade squamous intraepithelial lesions, squamous cell carcinoma, atypical glandular cells, endocervical adenocarcinoma in situ, adenocarcinoma).

^e^ Communicated in 14 days or less (n = 11 784).

^f^ Communicated in 30 days or less (n = 20 156).

^g^ The SHEP item evaluated was the question “in the last 6 months, when this provider ordered a blood test, x-ray, or other test for you, how often did someone from this provider’s office follow up to give you those results?” The SHEP median (range) was a January to December 2019 facility variation, and 1 facility was excluded because of insufficient sample size.

The sample included data from October 2018 to September 2019 for all 141 VA facilities. EPRP measures examined timeliness of communication to patients with abnormal results (≤7 days if action required; ≤14 days if no action required), normal results (≤14 days), and all test results (≤30 days). Additionally, we evaluated 1 item from the Survey of Healthcare Experiences of Patients (SHEP) during the same timeframe (ie, “in the last 6 months, when this provider ordered a blood test, x-ray, or other test for you, how often did someone from this provider’s office follow up to give you those results?”).

We used descriptive statistics to examine EPRP and SHEP data and a Pearson correlation between the EPRP measure for all test results and the SHEP item. Using Stata version 15.1 (StataCorp), statistical analysis was conducted using a significance threshold of *P* < .05 with a 2-tailed test. Data were given to us between February and May 2021 and were analyzed between March and November 2021.

## Results

EPRP measures showed timely communication for 5925 of 8372 abnormal results (70.8%) (ie, within 7 days if action was required and within 14 days if no action was required); for 9472 of 11 784 normal results (80.4%) (ie, within 14 days); and 16 479 of 20 156 (81.8%) of all test results within 30 days. Performance varied by facility; timely communication ranged between a median (range) of 71.4% (45.5%-93.9%) for abnormal results, 81.3% (52.9%-96.8%) for normal results, and 82.3% (58.5%-94.8%) for all tests (Figure). Performance also varied by test, for example, communication was timely the least often for dual-energy x-ray absorptiometry scans (512 of 828 [61.8%]) and most often for chest x-rays (477 of 560 [85.2%]) for abnormal tests (Table).

**Figure.**
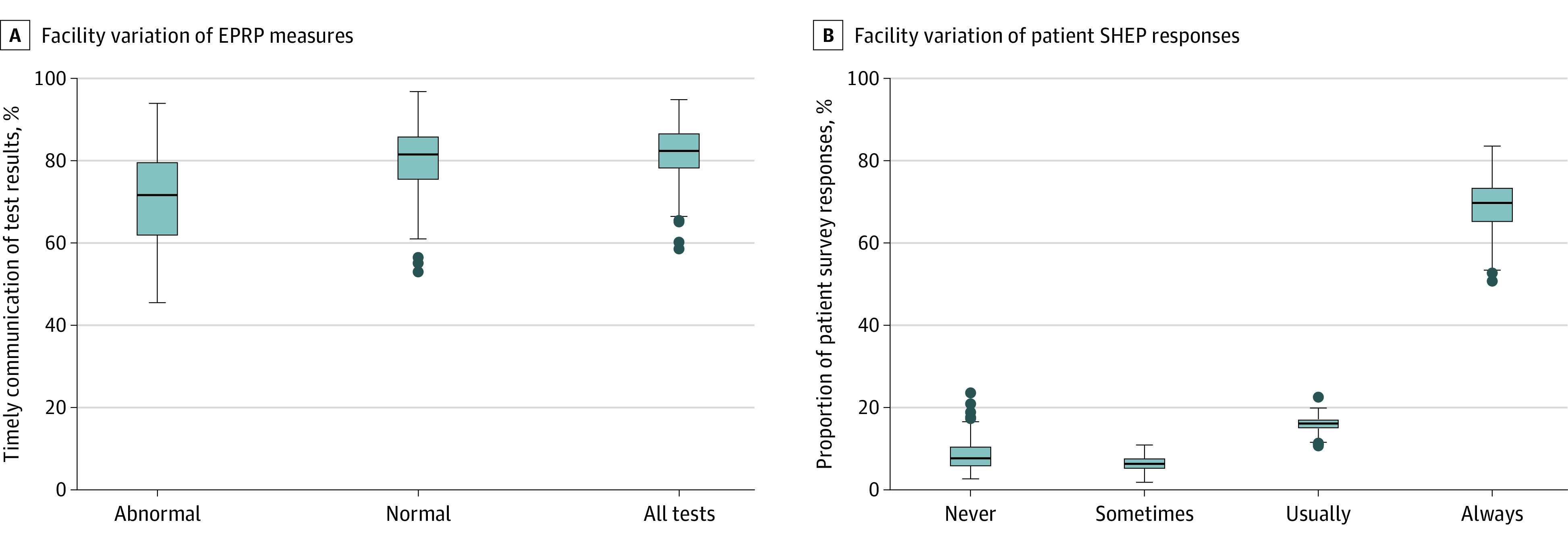
Variation in Timely Communication of Test Results to Patients Between Department of Veterans Affairs (VA) Facilities Box plots show variation in timely communication of test results to patients between VA facilities as measured by VA’s External Peer Review Program (EPRP; A) and the Survey of Healthcare Experiences Program (SHEP; B). Boxes represent IQR, horizontal lines in each box represent the medians, whiskers represent 1.5 times the IQR, and dots represent outliers.

In SHEP data from 213 464 patients, 17 435 patients (8.2%) said that results were never communicated, 13 456 (6.3%) said results were sometimes communicated, 33 673 (15.8%) said results were usually communicated, and 148 900 (69.8%) said results were always communicated. These findings varied by facility; for example, a median (range) of 7.6% (2.6%-23.5%) of patients reported results were never communicated, while a median (range) of 69.7% (50.7%-83.5%) of patients reported results were always communicated (Figure). The SHEP data of the facilities (ie, percentages of patients responding results were always reported) and EPRP measure of timely communication of all results were significantly correlated (*r* = 0.30; *df* = 128; *P* < .001).

## Discussion

The VA’s national performance measurement system reveals gaps in timely communication of test results to patients. Communication gaps varied by facility, emphasizing the need for local quality improvement efforts to address contextual factors impacting follow-up (eg, local workflows or team support for test result management).^6^ A limitation of this study is that these data rely on documentation and do not reveal all aspects of communication quality. Despite this limitation, the data are supported by patient experience surveys. The 21st Century Cures Act requires the release of test results to patients, but communication for abnormal results requires monitoring to ensure adequate follow-up. Similar quality measures can be implemented in other health systems able to query and review electronic health record data. Given persistent care gaps, these measures should be used as accountability metrics to facilitate wider implementation of known interventions to improve the timeliness of test results communication to patients.
